# Oral cavity and oropharyngeal squamous cell carcinoma in young adults: a review of the literature

**DOI:** 10.2478/raon-2013-0057

**Published:** 2014-01-22

**Authors:** Ewa Majchrzak, Bartosz Szybiak, Anna Wegner, Piotr Pienkowski, Jakub Pazdrowski, Lukasz Luczewski, Marcin Sowka, Pawel Golusinski, Julian Malicki, Wojciech Golusinski

**Affiliations:** 1 Department of Head and Neck Surgery, The Greater Poland Cancer Centre, Poznan, Poland; 2 Poznan University of Medical Sciences, Poznan, Poland; 3 The Greater Poland Cancer Centre, Poznan, Poland

**Keywords:** head and neck cancer, squamous cell carcinoma, young adults, quality of life

## Abstract

**Background:**

Head and neck squamous cell carcinoma (HNSCC) is a disease of middle-aged to elderly adults. However, an increased incidence of HNSCC in young people under 45 years of age has been reported recently. In the present review, we focused on the epidemiology and aetiology of HNSCC in adults under 45 years of age.

**Methods:**

We reviewed literature related to HNSCC in adult patients less than 45 years of age and discussed current treatment options and prognosis.

**Results:**

HNSCC in young adults is associated with a higher incidence rate in nonsmokers, lower female-to-male ratio, a higher percentage of oral cavity and oropharynx tumours, and fewer second primary tumours. However, aside from traditional risk factors of tobacco and alcohol exposure, the causes of these cancers in young adults remain unclear. Agents that might contribute to risk include infection with high-risk human papillomavirus subtypes as well as genetic factors or immunodeficiency status. The expected increase in incidence and mortality of the young with HNSCC may become a major public health concern if current trends persist, particularly lifestyle habits that may contribute to this disease.

**Conclusions:**

Given the younger age and potential long-term adverse sequelae of traditional HNSCC treatments, young adults should be treated on a case-by-case basis and post-therapy quality of life must be considered in any treatment-decision making process.

## Introduction

Squamous cell carcinoma of the head and neck (HNSCC) is primarily a disease of older adults, occurring most frequently in patients older than age 45. Epidemiological studies over last 20 years have shown a steady rise in the incidence of these cancers in younger adults (age 18–45 years), especially in cancers of the oropharynx and oral cavity.^1^,^2^ The predilection for these particular subsites *vs*. other sites such as the larynx or the hypopharynx remains unclear. Likewise, the aetiology for early onset of these neoplasms is not well understood.

Many conflicting reports have been published on the aetiology, natural history, and prognosis of HNSCC in young adults since this disease was recognized as a distinct clinical entity in youngers in the year 1974.^3^ In contrast to the “typical” patient with HNSCC, younger patients often do not present the traditional risk factors of alcohol and/or tobacco exposure.^4^ This leads us to suspect that other potential agents, such as inherent genetic factors, viral infections, and behavioural risk factors may be involved.

Numerous early reports of squamous cell carcinoma (SCC) concluded that the disease was more aggressive and the prognosis poorer in young adults *vs*. older adults.^5^–^7^ However, findings from more recent studies, such as those by Gilroy *et al*., Goldenberg *et al*. or Hafkamp *et al*. have not found any significant differences in outcomes between different age groups.^8^–^10^ Recently, superior survival of younger patients with oropharyngeal SCC was found to be related to a high-risk human papillomavirus (HPV) infection.^11^

Nevertheless, due to differences in patient’s age (younger or older than age 45), aetiology and tumorigenetic process or prognosis, we must at least consider the possibility that different groups may require different treatment approaches. This is especially true given the fact that the conventional treatment (*i.e*., surgical resection and adjuvant radio[chemo]therapy) can be functionally debilitating in young adults and may cause long-term adverse *sequelae*.

In the present study, we review the available literature on this topic and discuss key considerations in the treatment of HNSCC in patients under age 45.

## Epidemiology

HNSCC is an anatomically heterogeneous group of neoplasms arising from the mucosal surface of the oral cavity, oropharynx, hypopharynx, larynx, sinuses and other sites within the upper aerodigestive tract. The global incidence and mortality rates for HNSCC are 540 000 and 271 000 annually, respectively.^12^ In most countries, incidence and mortality rates have either remained stable or increased during the past four decades. Many studies have reported that, since the 1960s, the international incidence of HNSCC (particularly of the oral tongue and oropharynx), has increased in young adults.^13^ Surprisingly, this increase has occurred concurrently with a decreasing prevalence of cigarette smoking in the general population; importantly, this observation would not be expected if the only primary risk factors for all HNSCC were alcohol and tobacco abuse.^4^

Despite the fact that, the SCC in the oral cavity (OC) and oropharynx (OP) is traditionally regarded as a disease of the elderly, the incidence of OCSCC and OPSCC in patients under age 45 increases and accounts for approximately 1–6%.^14^,^15^ In countries where betel quid is frequently chewed, such as in Taiwan, young patients account for 16% and 28% of all oral tongue cancer patients.^16^ In fact, evidence by Gupta suggests that oral cancer may now be considered a “new epidemic”, as incidence rates are reaching high proportions possibly due to the availability of manufactured areca nut products.^17^ This rising incidence is most strongly seen in developing countries in South and Southeast Asia, where oral carcinoma is often the first or second most common site for malignant cancer.^18^ In Western countries, over the past 30 years, the incidence of OCSCC has been decreasing, while the frequency of OPSCC has been noted to increase.^1^

The majority of research on the changing epidemiology has focused on the HPV and its association with HNSCC, particularly in primary tumours of the oropharynx. An analysis of Swedish Cancer registry data (1958–1996) showed that husbands of women with cervical carcinoma had a significantly increased risk of developing either base of tongue or tonsil cancer.^19^ In the United States (U.S.), rates of HPV-related oropharyngeal SCC increased in the period from 1973–2004, especially for tonsillar cancer.^1^,^20^ In Australia, the incidence of HPV-related sites in the oropharynx increased by 1% per year between 1982 and 2005 in men and women.^21^

Interestingly, although the rate of OCSCC is observed to be decreasing in young individuals, the incidence of oral tongue squamous cell carcinoma (OTSCC) has been rising especially in young white women, age 18–44 years^22^, what is more surprising given the fact that OCSCC, unlike OPSCC, are not typically associated with the HPV infection (approximately 50% of patients with OPSCC and less than 20% of patients with OCSCC are positive for HPV16 DNA).^23^ Consequently, young white women form a unique subgroup of patients with no traditional risk factors of tobacco and alcohol abuse and who can not be associated with HPV infection.^22^ Presumably, other environmental exposure, genetic abnormalities, and other oncogenic viral infections must play an essential role in the oncogenesis process.

Both oral cavity and oropharyngeal cancers are more common in patients of African descent, as Slotman *et al.* reported in a study carried out in the U.S. Of patients under age 45, African-Americans accounted for 13% of oral cavity cancers *vs*. only 3% for white patients. For oropharyngeal cancers, the results were similar, with young African-Americans accounting for 15.3% of diagnoses *vs*. only 2% of young white patients. Slotman *et al.* also noted a lower 5-year survival rate for African-Americans in all age groups.^24^ The poor survival, particularly in black Americans has been attributed to differences in socioeconomic status and more advanced stage of disease at presentation.^3^

Other locations of head and neck tumours like *e.g*. nasopharynx, larynx, and hypopharynx constitute a rather rare and distinct group of neoplasms in patients less than 45 years of age. For example, according to the literature in the U.S. and Europe, the annual incidence of nasopharyngeal cancer in people younger than 30 years is estimated to range from 1 to 2 per million, and African-Americans are at higher risk.^25^,^26^ However, it is still more common in older adults than in younger ones. Moreover, the above-mentioned heterogeneous group of malignancies is characterized by quite different biology and aetiology factors than oropharyngeal and oral cavity cancers. That is why they are not further analyzed.

## Aetiology

### Tobacco and alcohol

Tobacco and alcohol have long been implicated as the traditional risk factors for HNSCC in adults, regardless of age. Individuals who smoke more than 20 cigarettes a day and consume more than 100 g of alcohol a day are believed to be at increased risk for oral epithelial dysplasia.^27^ In addition, alcohol has been found to be an independent risk factor for OCSCC among non-smokers and tobacco smoke in non-drinkers.^28^ Moreover, both factors together seem to enhance the carcinogenic effect.

Interestingly, many patients under age 45 declare never having smoked or consumed alcohol excessively, as Kuriakose *et al.* reported. Moreover, it has been suggested that exposure to such carcinogens might be of too short a duration for malignant transformation to occur in younger patients.^29^ Nevertheless, Llewellyn *et al*. and Lipkin *et al*. have both found that many young patients are heavy smokers and drinkers prior to their 40^th^ birthday.^30^,^31^ According to the findings reported by these researchers, tobacco consumption for more than 21 years results in an elevated risk of oral cancer. Llewellyn, in fact, noted that tobacco use often begins during adolescence (in many cases before age 16), thus making it quite probable that many patients have accumulated more than 21 years of addiction, with the increased risk of cancer that this implies, before age 40.

The rising mortality and increasing incidence of cancer of the tongue amongst young patients in the U.S. has been attributed to the use of smokeless tobacco products.^32^ However, this possible etiological risk factors has not been confirmed by subsequent studies. For instance, one study reported that smokeless tobacco was not implicated in the increase in incidence of oral cavity SCC in the United Kingdom during last 30 years.^32^ In another study, Thomas and Wilson evaluated betel-quid chewing as a risk factor for oral cancer, and studies in India have examined the role of betel-quid with and without tobacco in oral cancer cases, concluding that adding tobacco to the betel-quid significantly increases the risk of developing malignancies.^33^

### Marijuana and HNSCC

The first epidemiological study showing that marijuana smoking elevates the risk of head and neck cancers was published in 1999.^34^ Since that time, several case studies have been published that suggest an association between marijuana smoking and head and neck cancers, respiratory cancers and oral premalignant lesions. However, the carcinogenicity of tetrahydrocannabinol (THC) – the major psychoactive ingredient in marijuana – is still not clear. The tar component of marijuana contains similar carcinogens to tobacco, but each marijuana cigarette may be more harmful than a tobacco cigarette due to the characteristics of marijuana smoking: greater inhalation of tar, longer retention of marijuana smoke, and greater volume of marijuana smoke inhaled.^35^

In studies focusing directly on the tumour development and growth, cannabinoids have been shown to have both tumorigenic and antitumor properties.^36^,^37^ Reports of young adults with oral cavity SCC and other respiratory tract cancers raised the question of whether marijuana use really contributes to these malignancies. For instance, Rosenblatt *et al.*, in a large, population-based study, found no association between marijuana use and oral cavity SCC risk.^38^ In contrast, Liang *et al*. found that moderate marijuana use was significantly associated with reduced risk of HNSCC, a finding that did not differ across tumour sites or by HPV-16 antibody status. Moreover, they observed that marijuana use modified the interaction between alcohol and tobacco, resulting in a decreased HNSCC risk among moderate smokers and drinkers, and that it also an attenuated risk among the heaviest smokers and drinkers.^39^ However, this inverse association still needs to be confirmed by further studies.

### Human papillomavirus

Cervical cancer is the most widely accepted human papilloma virus (HPV)-associated malignancy. Recently, epidemiological and molecular data have suggested HPV, especially type 16, to be an independent risk factor in the development of HNSCC.^40^ The anatomical structures of oropharynx, most of all base of tongue and tonsils, seem to be favoured. Approximately 50% of patients with OPSCC are positive for HPV-16 DNA. On the contrary to this finding, oral SCCs are not typically associated with HPV presence, what could be due to the fact that the epithelial tissue of oral cavity differs from that in oropharynx structures, and only 20% of individuals with OCSCC are HPV-16 DNA positive.^23^

**FIGURE 1.**
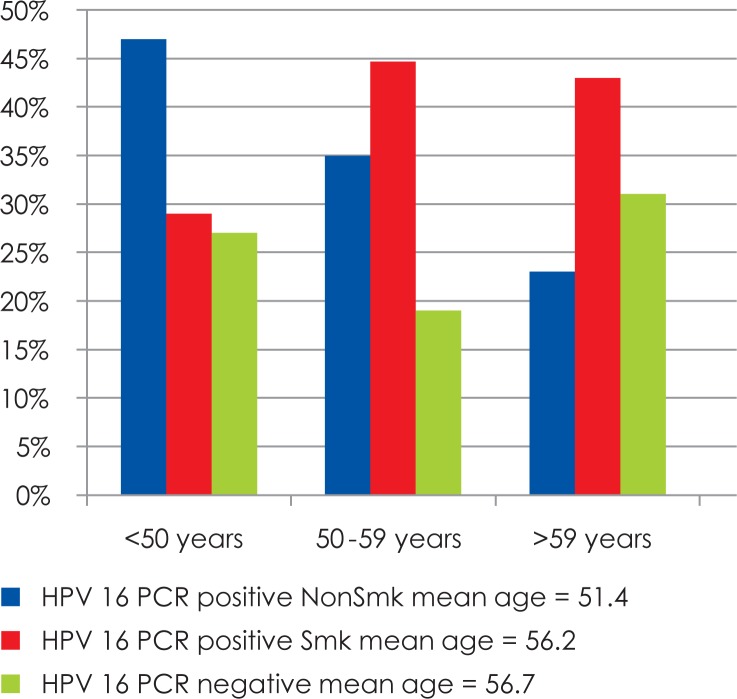
Oropharyngeal Cancer Patients (segregated by age, presence of HPV-16 infection and smoking).^41^

A strong association between HPV-16 positivity and oropharyngeal primary cancers was reported by Gillison *et al.* in a case control analysis.^41^ In addition, a higher proportion of these HPV-16 positive cases were young patients (Figure 1). A high number of lifetime vaginal and oral sexual partners, young age of onset of sexual activity, history of anogenital warts in men may be a potential source of viral colonization of the oral mucosa. However, patients with oropharyngeal SCC and higher numbers of sexual partners constitute only a small part of head and neck squamous cell carcinoma patients. Therefore, a low number of sexual partners does not exclude the diagnosis; husbands of women with *in-situ* and invasive cervical cancer, patients with a history of HPV-associated anogenital cancers, immunocompromised individuals (posttransplant patients and HIV infected ones) are also at high risk of developing HPV-associated HNSCC.^42^

**FIGURE 2.**
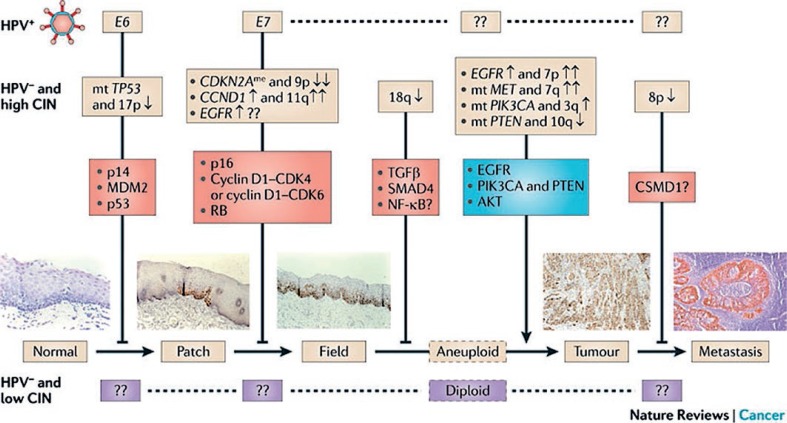
Proposal of an integrated model of molecular carcinogenesis for head and neck squamous cell carcinoma according to Leemans *et al.*^48^

Clinically, high risk HPV-related HNSCC tends to present with lymph node positive disease. Histologically, these neoplasms are usually high-grade and exhibit a basaloid morphology.^43^ On a molecular level, the HPV oncoproteins E6 and E7 bind with a high affinity to the p53 and retinoblastoma (Rb) tumour suppressor proteins, inducing their degradation (Figure 2). pRb is a negative regulator of p16 protein at the transcriptional level, with low pRb levels leading to subsequent p16 upregulation. Therefore, HPV-associated cancers are characterized with high p16 levels, low pRb and cyclin D1 protein levels, and wild-type p53 and pRb genes.^44^,^45^ On the contrary, typical for tobacco/ alcohol-associated head and neck cancers are downregulation of p16 protein, p53 gene mutation and overexpression of pRb and cyclin D1.^44^ Consequently, p16 overexpression proved to be a marker for oropharyngeal primary site and HPV-association.^46^

The incidence and clinical implications of biologically relevant HPV-16 infection through p16 protein expression in a cohort of OPSCC patients were studied at Yale University.^47^ The research resulted in delineation of three tumour classes with distinct molecular and clinical features on the basis of the presence of HPV-16 DNA and p16 expression status: HPV-16 negative/p16 nonexpressing (class I), HPV-16 positive/p16 nonexpressing (class II), and HPV-16 positive/p16 expressing (class III) oropharyngeal tumours. The multivariate survival analysis clearly showed that only HPV-16 posistive/p16 expressing tumours were associated with the favourable prognosis.

To summarize, HPV-related HNSCC patients constitute a unique population of patients who are typically younger, less likely to smoke and drink. These neoplasms usually exhibit a distinct biologic behaviour including improved response to (chemo)-radiation and survival when comparing to HPV-negative HNSCC. Moreover, because these patients do not smoke, there is often a delay in seeking medical care for their cancer related symptoms. More research is needed into the role of HPV in HNSCC, especially its connection to a treatment response.

### Human immunodeficiency virus (HIV) infection

Traditionally, the most common type of head and neck cancer in patients with HIV infection is Kaposi’s sarcoma and non-Hodgkin’s lymphoma. However, HNSCC occur frequently in this HIV-positive population. Recent publications have speculated whether the increased risk of HNSCC and lung cancer in HIV-infected populations is coincidental or related to the primary disease. Possible risk factors for carcinogenesis among these patients, apart from tobacco and alcohol exposure, include immunosuppression, opportunistic infections, and high-risk HPV subtypes.^49^,^50^

The relation between HIV infection and HPV-related HNSCC is complex. In a large population of HIV-seropositive and HIV-seronegative adults, Kreimer *et al.* found the prevalence of high-risk oral HPV infection greater in HIV-seropositive individuals (13.7% compared with 4.5%).^51^ Case-control studies of patients in the era prior to highly-active antiretroviral therapy (HAART) have suggested a younger age of diagnosis and a more aggressive clinical course in HNSCC patients with HIV infection. However, since the introduction of HAART, HIV-positive individuals with advanced aerodigestive tract cancer may now have a similar outcome as patients without HIV.^52^

### Genetic factors

It seems likely that there is a genetic predisposition for the cancer development at a young age, particularly in those patients with no recognized risk factors. It has been shown that patients younger than 30 years exhibit a significantly increased chromosome fragility following mutagen exposure when compared to older patients; it is thought that this fragility may lead to genetic abnormalities (associated with alterations in DNA repair genes).^53^ In addition, a higher frequency of microsatellite instability has been found in younger patients. Conversely, no significant differences between patients <35 years *vs*. patients > 75 years have been found in the expression of p53, p21, Rb and MDM2 proteins.^54^,^55^

When stratified by age, the younger cohort does not have the genetic alterations that are seen so consistently in older head and neck SCC patients. In fact, the mean number of aberrations in young non-smokers is less than 50% of that observed in older smokers.^32^ Moreover, Koch *et al*. found fewer genetic abnormalities in HNSCC of young non-smokers than in young smoking patients, results that imply that the genetic alterations in this group of patients are still unknown.^56^ Toner *et al*. performed molecular studies of young nonsmoking patients with HNSCC, finding that cancers in this group of patients is markedly different, not necessarily in any recognizable phenotypic way, but undoubtedly at the genetic level.^32^

Finally, family predisposition must be considered. Copper *et al*. and Foulkes *et al*. both found a significant relative risk of squamous cell carcinoma if first degree family members suffered from HNSCC, particularly so if the onset occurred before age 50, in which case risk increased more than two-fold in the case of siblings (Table 1, Table 2).^57^

### Other risk factors

Apart from the previously discussed risk factors for HNSCC, there are several other factors that may play an important role in cancerogenesis in the young, including chronic immunodeficiency states (Bloom syndrome, Wiskott-Aldrich syndrome)^58^, immunosuppression regimes following organ transplantation^59^, and anaemia occurring in Patterson Kelly/Plummer Vinson syndrome.^32^ Additionally, Fanconi anaemia, an autosomal recessive syndrome caused by defects in DNA repair, is associated with a high risk of developing malignancy at a young age (the incidence of HNSCC in this population is estimated to be 14% by age 40).^60^ Diets high in fruits and vegetables and fish oils are generally inversely correlated with a risk of oral cancer. Based on the studies by Llewellyn *et al*., this is also true for young adults.^13^

A distinct group of young patients with HNSCC consists of childhood cancer survivors. It is known that cancer patients have some risk of second synchronous or metachronous primary tumour. In 20-year survivors above-mentioned chance is estimated at the level of 3–12%. Chemotherapy drugs and radiation therapy are known for their long term carcinogenic effects; therefore, induced malignancies are one of the most serious side effects of the treatment of childhood cancer survivors.^61^,^62^

## Treatment and prognosis

HNSCC treatment recommendations and prognosis are currently based on TNM staging, status of the surgical margin, presence of lymph node extracapsular tumour spread and, in some instances, also on tumour differentiation, thickness, and presence of perineural and perivascular invasion. In the literature, many studies have commented on differences in stage between younger and older patients at the time of diagnosis.

Soudry *et al*. reviewed the 1992–2007 tertiary referral centre database and found young adults with oral tongue cancer to have a significantly worse clinical/radiological N stage at diagnosis and more evidence of perineural invasion on histopathological examination. However, those authors did not find any significant differences between younger and older patients in terms of histological grade, tumour depth, or presence of lymph node extra-capsular extension.^61^ Similarly, Veness *et al*. and Verschuur *et al*. found a higher incidence of nodal metastases in younger patients.^63^,^64^

Sturgis *et al.* indicated higher percentage of advanced HNSCC in young adults. According to their review, 73% of HNSCC were stage III or IV at presentation.^65^ In contrast, a research performed by Funk *et al.* reported that younger patients typically had an earlier stage of disease on presentation and, consequently, a higher proportion of stage I cancer was noted in younger age groups.^66^ Compared to HPV-negative patients, those with HPV-positive oropharyngel tumours have more frequently early-stage primary tumours and more advanced neck disease at the time of diagnosis.^11^

### Oral cavity squamous cell cancers

Traditionally, patients with HNSCC are treated with surgical resection and when indicated postoperative adjuvant radiotherapy. But such procedure may have a devastating effect on major functions like breathing, swallowing, speech and in consequence negative impact on the quality of the remaining life.^67^ As more and more people are surviving HNSCC also terms of appearance, function and shoulder mobility seem to be much more important.

Individuals, who develop HNSCC when young (40 years of age or less) and survive, create a different patient subgroup from the elderly people who develop cancer in their fifties through seventies. Young patients are usually healthy, active and have a long life expectancy.^68^

The local-regional control of oral cavity SCC has been increased mostly because of more aggressive surgical resection facilitated by modern reconstructive methods and advances in radiotherapy.^69^–^71^ Simultaneous postoperative chemoradiotherapy is believed to improve a local-regional control in patients possessing high risk features such as positive surgical margins and extracapsular tumour extension. However, distant recurrences still remain a problem in patients treated for oral cavity cancer. Also survival rates have improved only frugally over the past 3 decades.^72^

Considering the above data, new therapeutic options have been explored. Kies *et al.* performed a trial of induction chemotherapy followed by surgery for OTSCC in young adults (23 patients with OTSCC, T2–3, N0–2, M0).^73^ They believed that induction chemotherapy may have the potential to reduce the intensity and morbidity of subsequent local-regional treatment procedures (surgery and radiotherapy) and consequently increase quality of life. On the basis of pathologic review of the surgical specimen, 9 patients (39%) had a complete or major response at the tongue, 8 (35%) had no response or had progression of the primary tumour. In the neck, 9 patients (39%) had a complete response or remained node negative, and 6 (26%) had an increase in nodal stage. Distant recurrence rate of 30% observed in this trial raised assumption that induction chemotherapy selects the most aggressive subpopulations to survive, which resulted in distant recurrence.

Licitra *et al*. has also suggested that neoadjuvant chemotherapy may have a role in function preservation as well as in avoiding radiotherapy in younger patients with HNSCC, especially those with oral cancer.^74^ Similar findings have been reported by Sturgis *et al*., who have suggested that postoperative radiotherapy may not be necessary in some patients who undergo neoadjuvant chemotherapy.^65^ On the contrary to these observations, data presented at the American Society of Clinical Oncology (ASCO) 2012 annual meeting suggested no survival advantage of induction chemotherapy prior to chemoradiotherapy over chemoradiotherapy alone, which makes the role of neoadjuvant chemotherapy highly questionable.^75^,^76^

In many fields, the search for biological markers of disease is intense nowadays, and HNSCC is no exception. Molecular profiling of tumours has been driven by changes in epidemiologic patterns and the development of effective biologic agents directed against specific molecular targets. As Thomas *et al*. noticed EGFR overexpression in oral cavity tumours of young adults predisposes to a poor prognosis with a consequent adverse survival. Mixed results for OTSCC treatment with anti-EGFR antibodies have been presented in the literature. Nonetheless, EGFR overexpression may be a prognostic indicator in identifying patients who warrant a more radical approach to the treatment.^77^

### Oropharyngeal squamous cell cancers

At present, it remains speculative whether patients with HPV-positive HNSCC should be treated differently from those with HPV-negative tumors.^41^ Molecular profiling of HPV-positive tumours that are typically found in the oropharynx, has shown that these tumours seem to be commonly associated with p16 overexpression, whereas tumours not associated with HPV are seldom p16 positive. P16 positivity has been shown to be connected with improved outcomes, regardless of HPV infection status. Therefore, p16 positivity has been proposed to be a more reliable and reproducible prognostic marker in HNSCC.^78^ Furthermore, prognostic power of extracapsular tumour spread seems to be diminished in surgically treated p16-positive oropharyngeal SCC.^79^

However, the increasing recognition that HPV-related HNSCC are notably sensitive to radiation therapy has prompted investigators to question whether patients with HPV-associated HNSCC might be overtreated and unnecessarily subjected to the toxicity of intensive treatment strategies using chemoradiotherapy.^80^ HPV-positive HNSCC patients are consistently proved to have an improved prognosis when comparing to those with HPV-negative tumours. Moreover, it has been demonstrated by Chen *et al*. that clinical outcome among patients treated by radiotherapy alone for HPV-positive HNSCC appear to compare favourably to those treated by more intensive chemoradiotherapy approaches.^80^ Recently Ang *et al*. and O’Sulivan *et al*. identified group of patients, characterized by T1–3 and N0–2b HPV-positive oropharyngeal SCCs (in case of N2b disease, patients should be nonsmokers/minimal smokers) that would not necessarily need intensive chemoradiation and are candidates for treatment de-escalation clinical trials.^81^,^82^

The mechanism of HPV-mediated radio-response is unclear. The most direct explanation is that by the interference with the normal function of p53 and pRb, the viral products E6 and E7 render the host tumour cell more susceptible to radiation-induced apoptosis.^80^ This hypothesis was demonstrated by Pang *et al*. who showed that transfection of the E6 transcript in HPV-negative SCC cell lines resulted in sensitization to radiation-induced cell death.^83^

Although there are mainly clinical researches convicting much better prognosis for patients with HPV-positive HNSCC, it is still uncertain whether it is the improved radiosensitivity that drives the superior survival of these individuals. Namely, HPV-positive patients treated by surgery have also been shown to have better prognosis than HPV-negative ones.^84^

Two commercial HPV vaccines are available nowadays for the prevention of cervical cancer and genital warts: the quadrivalent vaccine Gardasil (Merck & Co. Inc., Collegeville, Pennsylvania, USA) targets HPV subtypes 6, 11, 16 and 18, and the bivalent vaccine Cervarix (GlaxoSmithKline, Research Triangle Park, North Carolina, USA) targets the subtypes 16 and 18. Both, they are able to elicit a robust immune response and in consequence significantly decrease the incidence of persistent HPV-16 and HPV-18 infections and associated moderate-to-high grade cervical neoplasia CIN2/3.^85^ Whether there is impact of these vaccines on the incidence of persistent oral HPV infection still must be identified.

## Treatment outcomes – the effect of patient’s age

Even though Byers first suggested as far back as 1975 that HNSCC in young adults should be considered a distinct subgroup, the question as to whether age has a significant impact or not on treatment outcomes still remains unanswered.^86^ However, several studies – *e.g*. von Doersten *et al.*^22^ and Funk *et al*.^66^ – have shown that patients under age 45 have a higher 5-year survival rate. Gilroy *et al*. found a significant difference in the overall survival in favour of younger patients, as did Verschuur.^8^,^64^ In fact, both of those studies reported similar findings in terms of cause-specific survival, locoregional control rates, and distant metastatic rates. Liao *et al*. evaluated 296 patients and found no differences in therapeutic outcome between young and older patients who had similar tumour characteristics, therapeutic modalities, and pathological risk factors. However, although they found no significant differences in local control rate or neck control rate, they did observe a higher rate of distant failure in young adults.^87^ Soudry *et al*. concluded that, in general, patients younger than 45 years have the same outcome as older patients.^61^ However, within the younger group two distinct patterns of disease were observed: an extremely aggressive course with a high mortality rate within 2 years and a more indolent course with a lower mortality rate. Veness *et al*. found a higher rate of locoregional recurrence in younger *vs*. older patients.^63^ In contrast, Van Doersten *et al*., in a multivariate analysis of 155 patients, found that age had no effect on recurrence rates.^22^ Verschuur concluded that younger patients had a significantly lower incidence of second primary cancers compared with an older cohort.^64^ In contrast, Friedlander *et al*. reported the incidence of a second primary tumour to be similar between younger and older patients, and with no difference between groups in tobacco and alcohol use.^88^

## Conclusions

Many controversies still surround HNSCC in young adults. An important and still unanswered question is whether HNSCC in the young is a distinct clinical entity. Moreover, doubts about differences in etiologic risk factors between younger and older patients are still considerable, as are questions about the possible influence of younger age on prognosis. Moreover, the relatively low incidence of HNSCC in young adults hampers progress as it is difficult to perform studies and reach meaningful conclusions due to the limited numbers of patients.

Nevertheless, one thing is clear. Although young people have a lower incidence rate for HNSCC, physicians need to be aware that the incidence is growing and these types of cancers must be suspected in any patient with worrying signs and symptoms, regardless of age.

## Figures and Tables

**TABLE 1.** Suspicion of familial predisposition.^57^

**When familial predisposition is suspected?**
First degree relative with same or related cancer with other features in common
Two or more first degree relatives with same cancer, rare cancer
Two or more relatives in 2 or more generations with tumors of the same site

**TABLE 2.** Features of familial cancer syndromes.^57^

**Familial cancer syndromes**
Increased frequency
Shorter latency
Supporting events
Increased aggressiveness and treatment resistance Multiple primaries
Involved genes often mutated in sporadic cancers from the same site
